# Impact **of** distilled water dealkalization **on the** geotechnical behavior **of** red mud

**DOI:** 10.1371/journal.pone.0334002

**Published:** 2025-10-08

**Authors:** Ömür Çimen, Halil İbrahim Günaydın

**Affiliations:** 1 Department of Civil Engineering, Faculty of Engineering and Natural Sciences, Süleyman Demirel University, Isparta, Türkiye; 2 Department of Construction, Akdeniz University, Antalya, Turkey; Maria Curie-Sklodowska University: Uniwersytet Marii Curie-Sklodowskiej, POLAND

## Abstract

Red mud (RM), a highly alkaline residue from alumina production, poses environmental risks and limits its use in geotechnical applications due to its high soluble alkali content. This study investigates the effect of distilled water washing as a practical dealkalization method and assesses its impact on the geotechnical properties of RM. Both untreated and dealkalized samples were systematically characterized by chemical, mineralogical, microstructural, and geotechnical methods. The washing process reduced soluble alkali oxides while maintaining the primary crystalline phases, with only minor changes in diffraction peak intensities. SEM and BET analyses revealed particle agglomeration and alterations in mesoporosity, reflecting modifications in surface characteristics. These changes were consistent with a shift in particle size distribution and were accompanied by modifications in compaction characteristics, permeability, compressive strength, and swelling behavior, while plasticity indices showed only minor variations without altering the soil classification. The findings indicate that distilled water dealkalization is associated with measurable changes in the geotechnical response of RM by reducing soluble alkali ions while preserving the primary mineral phases and overall chemical composition. By relating the observed geotechnical changes to accompanying chemical and microstructural results, the study provides one of the few comprehensive evaluations of dealkalized RM, addressing an important gap in understanding its engineering potential.

## Introduction

The exponential growth of urban populations, coupled with increasing living standards and industrial expansion, has precipitated a marked escalation in waste generation across multiple sectors. Among these, industrial waste from mining and metallurgical processes poses a critical environmental and economic challenge [1]. One notable example is red mud (RM), a highly alkaline by-product generated during the Bayer process for alumina extraction from bauxite ore. Its composition—rich in iron, titanium oxides, sodium aluminium silicates, and other metal oxides—varies significantly depending on the geological origin of the bauxite and the specific process conditions applied [2,3]. The detailed elemental distribution of RM is summarized in Table 1 [3,4].

**Table 1:** Typical concentration ranges of major oxides and trace elements in RM.

Major Components	Weight (%)	Trace Elements	Concertation (mg/kg)	Trace Elements	Concertation (mg/kg)
Fe_2_O_3_	5-55	U	50-60	Mn	85
Al_2_O_3_	10-50	Ga	60-80	Y	60-150
SiO_2_	2-45	V	730	Ni	31
Na_2_O	0-10	Zr	1230	Zn	20
CaO	2-14	Sc	60-120	Lanthanides	0,1%−1%
TiO_2_	1-15	Cr	497	Th	20-30

Globally, RM is produced at a rate of 1 to 1.5 tonnes per tonne of alumina extracted, resulting in an annual output of approximately 150 million tonnes and cumulative stockpiles exceeding 4.6 billion tonnes [5,6]. This waste poses long-term environmental risks due to its high alkalinity, radioactivity, and metal content [7]. Despite improvements in dry stacking techniques [8], serious containment failures still occur. A defining case was the 2010 Ajka disaster in Hungary, where approximately 1 million m³ of RM inundated a 45 km² area, resulting in human casualties and widespread contamination [9]. More recently, in 2018, intense rainfall triggered an overflow from the Hydro Alunorte refinery’s containment system in Brazil, discharging highly alkaline bauxite residue into the Pará River and surrounding communities in the Amazon region [10]. While these cases directly involved RM, other significant tailings failures underscore the systemic fragility of containment infrastructure more broadly. For instance, the 2019 Brumadinho dam collapse in Brazil resulted in 259 fatalities and over 10 million m³ of mining waste released [11], while the 2022 Jagersfontein disaster in South Africa unleashed over 6 million m³ of alkaline, metal-laden slurry from historic diamond processing [12]. Several more recent events—including the 2024 containment breach in Chile’s Valparaíso Region and the 2025 dam collapse in Chambishi, Zambia—further illustrate the environmental and structural risks associated with tailings storage systems under diverse operational conditions [13,14]. These examples highlight not only the persistent vulnerabilities of such infrastructure but also the need for scalable and sustainable reuse strategies to reduce long-term hazards [15].

Increasing environmental pressures have spurred global interest in sustainable alternatives to traditional landfill-based waste management. Recycling, reuse, and valorization of industrial waste are increasingly acknowledged as essential strategies; however, their practical implementation remains limited [16]. In the aluminum industry, the most viable alternative to RM disposal is the reuse of RM as a secondary resource. This approach also offers the advantage of reducing disposal costs and potential pollution issues. Consequently, studies on the utilization of RM have garnered increasing interest in recent years. RM has been proposed for use primarily as a component or additive in construction materials and ceramics, as an adsorbent for toxic pollutants, and as an amendment for stabilizing heavy metals and metalloids in contaminated soils [17]. Nevertheless, its high alkalinity poses a significant challenge to the safe and efficient reuse of the material in an environmentally responsible manner [18]. A high pH, as determined by a set of standard test criteria, can present a potential issue in any given application. Any material with a pH greater than 11.5 is typically considered hazardous [2]. Given its caustic nature and high pH, RM represents a significant environmental hazard, particularly in areas in close proximity to disposal sites. This is a significant issue that must be addressed when considering the reuse of RM. It is therefore imperative to reduce the contamination risk associated with this material. Numerous research initiatives have concentrated on alleviating this adverse impact, with the majority of studies focusing on reducing the alkalinity of RM through dealkalization treatments [19]. Adjusting the pH of RM to around 9 has been widely recognized as an effective strategy for mitigating its environmental risks [20].

The alkali content in RM is primarily present in two forms: (i) soluble alkali compounds, including NaOH, Na₂CO₃, NaAl(OH)₄, and NaAlO₂, which release Na⁺ and OH⁻ ions into solution and can be effectively removed through water washing; and (ii) chemically bound alkalis that are incorporated into insoluble mineral phases such as cancrinite, sodalite, tricalcium aluminate, or hydrogarnet-type compounds [21–23]. Effective dealkalization necessitates either the conversion of bound alkalis into soluble forms for extraction or the stabilization of soluble alkalis into less mobile, insoluble forms through precipitation or ion exchange [24]. Consequently, dealkalization strategies are generally classified into three primary categories: physical methods (primarily water washing) [25–27], chemical methods (e.g., acid neutralization [28–30], CO₂ carbonation [31,32], gypsum treatment [33,34], seawater washing [35,36]), and biological approaches (e.g., microbial acid production) [37,38], each targeting specific alkali forms or transformation pathways [39].

Among the aforementioned methods, water washing has emerged as the most commonly employed physical approach due to its operational simplicity, environmental safety, and cost-effectiveness [28,40]. It facilitates the removal of readily soluble alkali ions, particularly Na⁺ and OH ⁻ , without introducing secondary chemical agents into the system. Although this method is less effective in targeting structurally bound alkalis, it serves as a fundamental step in preliminary dealkalization, especially in large-scale or resource-limited settings [41]. Numerous studies have examined the efficacy of water-based dealkalization methods under various experimental conditions, including differing liquid-to-solid (L/S) ratios, washing cycles, temperatures, and durations. Table 2 presents a summary of selected studies employing purified water to remove soluble alkalis from RM. These investigations collectively demonstrate that while water washing effectively reduces free alkali content and pH levels, its performance is significantly influenced by process parameters such as contact time, the number of cycles, and solution volume. For example, Li et al. [42] documented a progressive enhancement in dealkalization efficiency with extended contact time, achieving 12.52% after 120 minutes. Similarly, Kinnarinen et al. [39] identified a plateau in Na⁺ and OH⁻ removal beyond an L/S ratio of 7, suggesting limited advantages from additional solvent. In contrast, Zeng et al. [27] attained over 84% removal at 90 °C with an increased L/S ratio and extended leaching duration. Notably, it has been shown that at room temperature, a liquid-to-solid ratio of 5:1, and after one day of soaking and five washing cycles, up to 75% of the free alkalis in RM can be removed. However, the chemically bound alkalis remain largely unaffected by such treatments [44]. Collectively, these findings underscore the critical role of contact time and solvent volume in governing the efficiency of water-based dealkalization, while also highlighting the inherent limitations of this method in addressing more persistent alkaline phases.

**Table 2:** Summary of water-based dealkalization studies on RM under varying experimental conditions.

Reference	L/S Ratio (mL/g)	Dealkalization Method	Dealkalization Outcome
Zhu et al. (2015) [5]	3-5-7-9	Distilled water leaching (1x)	Marginal improvement beyond L/S = 7
Kinnarinen et al. (2015) [39]	2-4-6-8-10	Millipore water leaching (1x)	Na⁺ and OH⁻ concentrations plateaued beyond L/S = 6–7
Li et al. (2017) [42]	7	Distilled water leaching (1x, 30-60-90-120 min)	Dealkalization rate increased with time, reaching 12.52% at 120 min
Li et al. (2018) [25]	1-2-3-4-5-6-7	Deionized water leaching (1x, 23 h)	Dissolved alkalis increased with L/S ratio, with the sharpest increase at L/S = 2 and diminishing returns beyond L/S = 5
Tsamo et al. (2019) [26]	75	Distilled water soak (10 d)	pH dropped from 10.77 to 8.69 with nearly complete Na₂O removal
Zeng et al. (2022) [27]	4-6-8-10	Distilled water leaching (1x, 20-40-60-80-100 min, 90°C)	An Na₂O removal efficiency of 84.04% was achieved at a L/S ratio of 8 mL/g, with no further improvement observed at L/S = 10
Zhang et al. (2023) [40]	1	Deionized water leaching (8x, 2 h)	Eight sequential leaching cycles resulted in 17.0% Na₂O reduction and a pH decrease from 10.8 to 9.4
Rui et al. (2024) [43]	5	Distilled water leaching (30x, 24 h)	A sharp decline in Na⁺ concentration and a pH drop (from ~10.8 to ~9.3) were observed within the first two washing cycles, with both parameters gradually stabilizing after the 10th cycle
Liu et al. (2025) [32]	4	Distilled water leaching (30x, 24 h)	A substantial decline in Na⁺ concentration was observed during the first five washing cycles, followed by only marginal decreases in subsequent stages

Among the various dealkalization strategies developed for RM—including acid leaching, seawater treatment, CO₂ carbonation, and gypsum addition—distilled water washing remains one of the most operationally practical and environmentally benign approaches. Distilled water washing not only removes readily soluble alkaline and disrupts lattice-bound alkalis without introducing secondary ions that might interfere with subsequent physicochemical or geotechnical evaluations, but also enhances the physical handling of red mud by increasing settling rates, improving particle size distribution, and reducing surface charge [26]. While previous research has primarily focused on the chemical effects of distilled water treatment, such as pH reduction and ionic mobility [25,27,32,39,40,42,43], its implications on the geotechnical performance of RM remain underexplored. Addressing this gap, the present study investigates the effects of distilled water dealkalization on the physical, mechanical, and structural behavior of RM sourced from the Seydişehir Eti Aluminum plant in Turkey. A series of experimental tests—covering specific gravity, particle size distribution, Atterberg limits, compaction, permeability, unconfined compressive strength, and free swelling—were conducted to evaluate changes in geotechnical behavior. These were supported by chemical (XRF), mineralogical (XRD), and morphological (SEM) analyses to capture the underlying structural transformations induced by dealkalization. This systematic investigation offers new insights into the engineering response of RM to water-based treatment, and contributes toward a more informed assessment of its reuse potential in geotechnical applications.

## Experimental

### Materials

**Fig 1:**
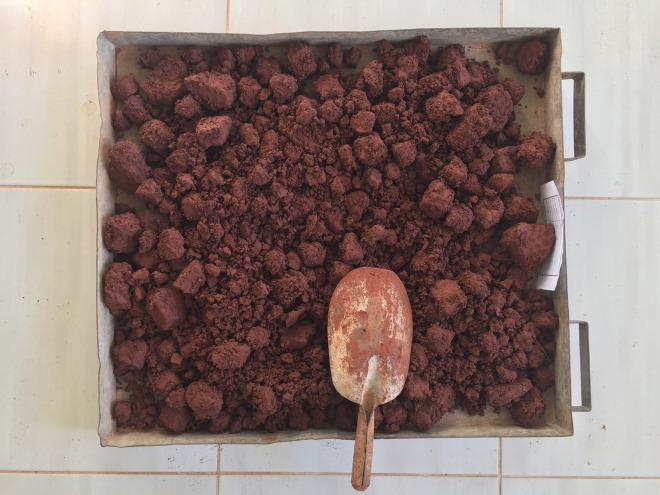
Field-collected wet RM samples.

The RM used in this study was supplied by the Seydişehir Eti Aluminum plant located in the Seydişehir district of Konya, Turkey. This facility processes approximately 500,000 tons of bauxite annually, yielding between 500,000 and 750,000 tons of RM as a by-product. This waste material is stored in tailings dams in proximity to the factory. In the past, RM slurry, containing approximately 30% solids, was transported to dams. However, in recent years, press filters have been employed to increase the solid content to approximately 70%, thus improving the efficiency of the dam storage capacity [45,46]. Upon receipt in the laboratory, RM samples were stored in large containers and thoroughly mixed to ensure homogeneity. Subsequently, the samples were air-dried at room temperature. The required amount of material for the experiments was obtained using the quartering method in accordance with EN 932−2 [47] (Fig 1).

### Sample preparation and dealkalization

**Fig 2:**
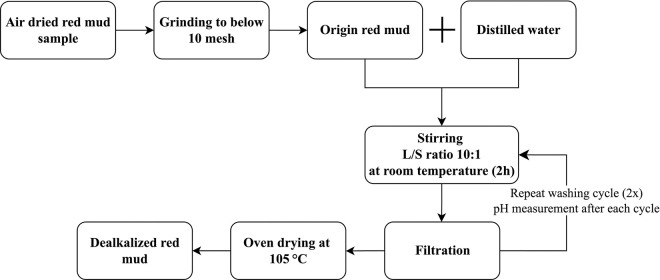
Experimental procedure for RM dealkalization.

A two-step dealkalization procedure was employed on the RM samples using distilled water. Initially, the air-dried RM was gently ground using a mortar and pestle until it could easily pass through a No. 10 mesh (2 mm), thereby removing coarse agglomerates that might impede the dissolution efficiency during the subsequent dealkalization process. In each dealkalization cycle, the RM was suspended in distilled water at a L/S ratio (mL/g) of 10:1 and continuously stirred at room temperature for 2 hours. Following the first cycle, the suspension was filtered, and the process was repeated under identical conditions with freshly replaced distilled water to enhance the removal efficiency of soluble alkali and prevent re-adsorption or ionic equilibration with the previously extracted species. Although the procedure primarily aimed to reduce the high pH of RM, the underlying mechanism involved the progressive leaching of soluble alkali ions (mainly sodium) into the aqueous phase over multiple washing cycles [24]. The pH of the RM was determined using its aqueous extract, which was prepared by mixing the dealkalized sample with distilled water at a liquid-to-solid ratio of 2.5:1 (v/w). Following thorough mixing and subsequent settling, the supernatant was separated by filtration and its pH measured using a calibrated pH meter with an accuracy of ±0.01. The experimental procedure for the dealkalization process is depicted in Fig 2.

### Characterization and testing methodology

To investigate the influence of distilled water dealkalization, untreated and dealkalized RM samples were characterized through chemical, mineralogical, microstructural, and geotechnical analyses. In preparation for characterization and testing, the samples were oven-dried at 105 ± 5 °C for 24 h in accordance with EN ISO 17892–1:2015 [48] and established practices in RM characterization [30,31,40,49,50], to remove free and adsorbed moisture. Chemical and mineralogical composition was assessed by XRF and XRD, microstructural features were examined by SEM-EDS, and surface area and porosity were quantified using BET analysis. Geotechnical characterization comprised Atterberg limits, specific gravity, grain size distribution, compaction, permeability, unconfined compressive strength, and free swell tests. All procedures followed the relevant EN and ASTM standards. Where applicable, tests were repeated to verify reproducibility, and variability was assessed where possible. Detailed procedures for each characterization and geotechnical test are provided in the subsections that follow.

#### Chemical, mineralogical, and microstructural analyses.

X-ray Fluorescence (XRF) Analysis: The major oxide composition of the samples were determined using wavelength-dispersive XRF (Rigaku ZSX Primus II) on pressed powder pellets, with primary oxides quantified as SiO₂, Al₂O₃, Fe₂O₃, CaO, MgO, Na₂O, K₂O, and TiO₂.

Scanning Electron Microscopy (SEM) and Energy Dispersive Spectroscopy (EDS) Analysis: Surface morphology and microstructural features were examined using a field emission gun scanning electron microscope (FEI Quanta FEG 250). Samples were coated with a thin carbon layer to improve conductivity, and imaging was carried out under low-vacuum conditions using secondary electron (SE) and backscattered electron (BSE) detectors. Elemental composition was assessed with an integrated EDS detector (EDAX), operated in area mode on representative regions of each specimen, with results expressed as semi-quantitative weight percentages (wt%).

X-ray Diffraction (XRD) Analysis: Phase composition and crystallinity were assessed using an X-ray diffractometer (Bruker D8 Advance Twin-Twin) with Cu-Kα radiation (λ = 1.54060 Å). Diffraction data were collected at room temperature across a 2θ range of 5°–85°, using a 0.05° step size and 0.1 s/step scan rate, with the X-ray tube operated at 40 kV and 30 mA.

Brunauer–Emmett–Teller (BET) Analysis: Specific surface area and pore characteristics were determined via nitrogen (N₂) adsorption at 77 K using a surface area analyzer (Micromeritics Gemini VII 2390t). Samples were degassed in two stages: first at 90 °C for 60 min, followed by 350 °C for 240 min under a vacuum of 1.0 × 10 ⁻ ² mmHg. Measurements were performed over a relative pressure (P/P₀) range of 0.05–0.25 to obtain adsorption isotherms and calculate surface area (m²/g), micropore area, and pore size distribution. The t-plot method was used to determine micropore and external surface areas.

#### Geotechnical tests.

Specific Gravity (G_s_): The specific gravity of RM was determined in accordance with EN 1097−6 [51] using a pycnometer on oven-dried samples.

Atterberg Limits: The liquid limit, plastic limit, and plasticity index were determined following EN ISO 17892−12 [52] on samples passing the No. 40 sieve.

Grain Size Distribution: The particle size distribution was analyzed using sieve and hydrometer methods as per EN ISO 17892−4 [53] using oven-dried samples. Material finer than the No. 200 sieve was examined hydrometrically.

Compaction Properties: Maximum dry density (MDD) and optimum moisture content (OMC) were determined using the Standard Proctor test in accordance with EN 13286−2 [54] on samples passing the No. 4 sieve and compacted with a 2.5 kg hammer dropped from 30.5 cm.

Permeability Properties: Hydraulic conductivity was measured using the falling-head method in accordance with EN ISO 17892−11 [55] on samples compacted to MDD and OMC with material passing the No. 4 sieve. The test results were expressed in terms of the coefficient of permeability (k) in cm/s.

Unconfined Compressive Strength (UCS): UCS was determined following ASTM D2166/D2166M-16 [56] on cylindrical specimens (38 mm diameter, 76 mm height) molded at MDD and OMC, loaded at a strain rate of 0.5 mm/min until failure.

Free Swell Properties: One-dimensional free swell tests were performed according to ASTM D4546-14 [57] using oedometer rings (71.5 mm diameter, 17 mm height). Swelling deformation and pressure were recorded under unconfined conditions until equilibrium.

## Result and discussion

### Effect of dealkalization on pH and chemical composition

**Fig 3:**
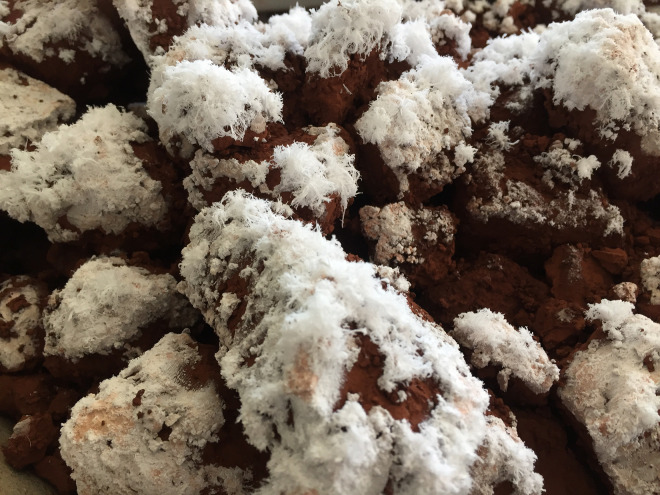
White crystal formations observed on the surface of air-dried RM samples.

The elevated initial alkalinity of RM, indicated by pH values surpassing 11, is predominantly due to the residual caustic soda (NaOH) from the Bayer process employed in alumina extraction [21]. This residual alkalinity is present in both free and chemically bound forms. Free alkali, which is readily soluble in water, contributes to the immediate high pH, whereas bound alkali, often integrated into minerals such as sodalite, serves as a more enduring source of alkalinity [20,24,40]. Upon exposure to air, carbonation reactions occur between atmospheric CO₂ and soluble alkaline species, resulting in a gradual decrease in pH [58]. In this study, air-dried samples exhibited the formation of white crystalline deposits (Fig 3), which were consistent with sodium carbonate (Na₂CO₃) precipitation due to the evaporation of alkaline effluents.

Following natural air-drying, the initial pH of the RM was recorded at 10.65, indicating the presence of residual alkalinity from caustic soda within the material. After the first washing cycle, the pH decreased to 9.80, and following the second cycle, it further declined to 9.35. This progressive reduction indicates the gradual release of free alkali ions, particularly Na⁺ and OH ⁻ , into the aqueous phase. These results align with the observations of Kinnarinen et al. [39], who identified pH reduction as a direct consequence of the dissolution of caustic species during aqueous dilution. Zeng et al. [27] emphasized the role of pH evolution as a diagnostic parameter for assessing the effectiveness of water-based dealkalization treatments. Li et al. [25] observed a reduction in pH from 10.47 to 9.57 following six consecutive washing stages with a L/S ratio of 2:1. This finding substantiates that cumulative dealkalization cycles under controlled conditions can result in substantial decreases in soluble alkalinity.

The alterations in the major compositions of the RM following the dealkalization process are presented in Table 3. Notably, the contents of Na₂O and K₂O decreased from 6.00% to 4.36% and from 0.19% to 0.12%, respectively, reflecting the dissolution and removal of soluble alkali salts during the two-stage water washing treatment. In contrast, the concentrations of CaO, Fe₂O₃, Al₂O₃, SiO₂, and TiO₂ remained largely unchanged, suggesting that the process selectively targeted mobile alkali species without significantly affecting the structural mineral framework. The observed trends align with those reported in previous studies. Ma et al. [23] identified key soluble free alkalis (SFAs)—including NaOH, Na₂CO₃, NaAl(OH)₄, and Na₂SiO₃—as readily dissociable in aqueous media, releasing Na ⁺ , K ⁺ , and OH⁻ ions. Similarly, Zhai et al. [59] differentiated between water-soluble alkali species and structurally bound alkalis embedded within stable mineral phases, such as sodalite and cancrinite. The observed reduction in Na₂O and K₂O confirms that the water-washing strategy effectively eliminated soluble phases, while structural alkalis likely remained intact. These observations are in agreement with mechanistic insights into alkali speciation in RM and further substantiate the viability of reagent-free, water-assisted dealkalization strategies in effectively mitigating reactive alkalinity [5,26,32,42,43].

**Table 3:** Major chemical components of untreated and dealkalized RM analyzed by XRF (wt.%).

	Chemical composition
	Na_2_O	K_2_O	CaO	Fe_2_O_3_	SiO_2_	Al_2_O_3_	TiO_2_
Untreated RM	6.00	0.19	13.67	27.91	13.87	21.98	3.17
Dealkalized RM	4.36	0.12	13.38	28.14	13.57	22.12	3.20

### Effect of dealkalization on structural and mineralogical properties

#### Microstructure transformation.

**Fig 4:**
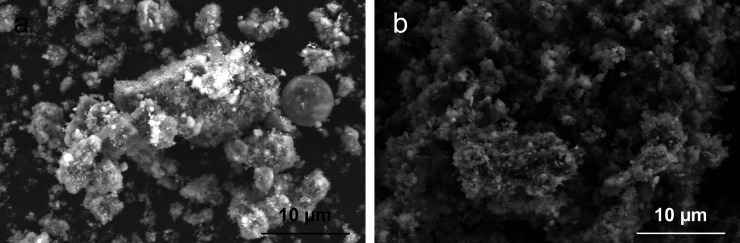
SEM micrographs of samples: (a) untreated RM, (b) dealkalized RM.

Fig 4 presents SEM micrographs of both untreated and dealkalized RM samples. The morphology of the untreated sample (Fig 4a) reveals irregularly shaped particles, comprising both angular and spherical grains, which frequently form coarse and loosely bound agglomerates. This disordered structure is typically associated with elevated residual alkalinity, which induces strong electrostatic interactions and impedes the formation of stable microstructures [5,6,27]. In contrast, the dealkalized sample (Fig 4b) exhibits a more compact and homogeneous structure, characterized by more closely packed and morphologically uniform particles. This transformation likely indicates alkali-induced reorganization, as the reduction of free alkali species (e.g., Na⁺ and K⁺) diminishes surface charge repulsion and promotes interparticle adhesion. Similar morphological consolidation has been documented in previous studies, where water-based or thermal treatments facilitated the emergence of aggregated microstructures and enhanced particle connectivity [27,43,60]. These findings support the view that reagent-free dealkalization can lead to local agglomeration and structural refinement, which are essential for the subsequent physicochemical behavior of RM in environmental or engineering applications.

**Fig 5:**
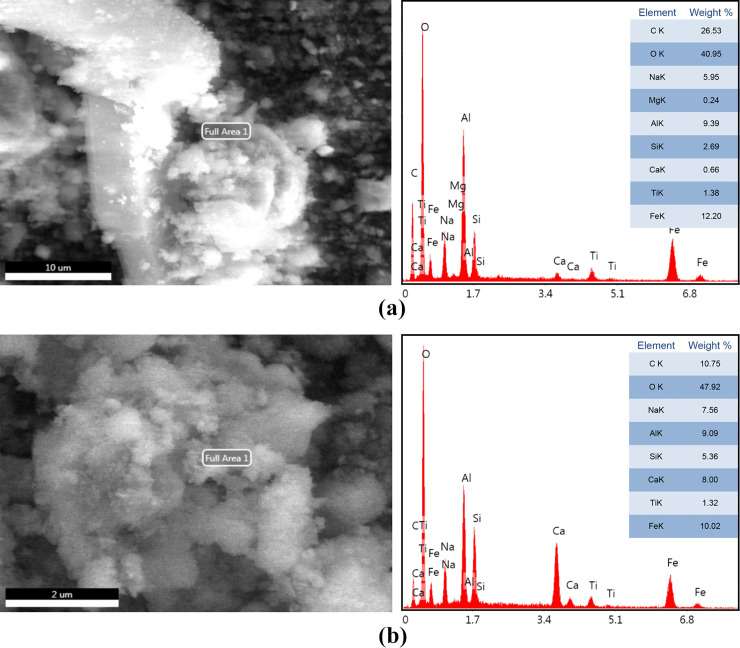
EDS analyses of samples: (a) untreated RM with the selected analysis area and spectrum, (b) dealkalized RM with the selected analysis area and spectrum.

The EDS area analyses for untreated and dealkalized RM are presented in Fig 5, where the selected analysis areas, corresponding spectra, and semi-quantitative elemental compositions are presented collectively. As illustrated in Fig 5a, the untreated RM spectrum indicates Fe, Al, and O as dominant elements, accompanied by Na, Si, Ca, and Ti. The spectrum of the dealkalized sample (Fig 5b) reveals a similar profile, confirming that the major constituents remain detectable after treatment. Changes in the relative intensities of Na, Si, and Ca peaks were evident, which likely reflect localized surface redistribution rather than bulk compositional change, consistent with the semi-quantitative and surface-sensitive nature of EDS. These results are consistent with previous studies [5,40,43], which reported that mild neutralization methods, such as water washing, generally preserve the bulk phase composition while inducing surface-level reorganization.

#### Surface area and pore structure.

**Fig 6:**
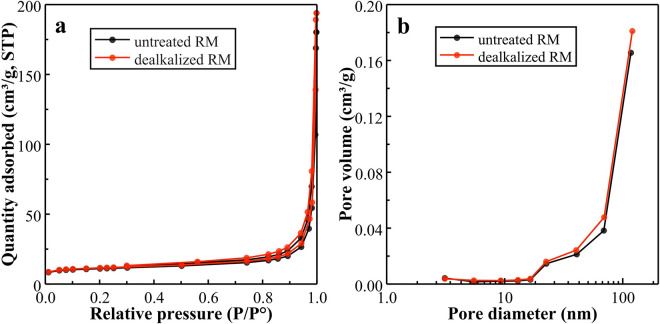
N₂ adsorption–desorption isotherms (a) and BJH pore size distribution curves (b) of untreated and dealkalized RM.

The N₂ adsorption–desorption isotherms and pore size distribution curves of untreated and dealkalized RM are presented in Fig 6. As seen in Fig 6a, both samples display curves consistent with type IV isotherms accompanied by H3-type hysteresis loops, typically associated with slit-like mesopores formed by the aggregation of plate-like particles [49,50]. The adsorption branch shows a gradual increase at lower relative pressures, with a moderate rise in the 0.3–0.9 p/p⁰ range, which may be related to capillary condensation within mesopores [61,62]. Such patterns are in close agreement with those reported in earlier studies on RM and its modified forms, which frequently exhibit type IV isotherms with H3-type hysteresis loops. These features have been commonly associated with layered particle morphologies and heterogeneous pore structures [ 50,63,64]. As illustrated in Fig 6b, the BJH pore size distribution curves suggest that both untreated and dealkalized RM may contain mesopores mainly between 3–50 nm, with a peak near 4 nm and a broader distribution extending into the macropore region above 40 nm. Comparable bimodal distributions have been reported for acidified [50] and thermally treated RM [65,66], where modification appears to increase mesopore volume and reduce average pore diameter. The dealkalized RM in this study likewise shows a slight shift toward smaller pore sizes and a marginal tendency toward higher mesopore volume, which may indicate improved pore accessibility without substantial changes in overall structure. These trends are in general agreement with earlier reports on RM composites [63,65].

The textural parameters of untreated and dealkalized RM, obtained from N₂ adsorption–desorption isotherm analysis using BET, t-plot, and BJH methods, are summarized in Table 4. Untreated RM exhibited a BET surface area (S_BET_) of 36.58 m²/g, a micropore surface area (S_micro_) of 20.50 m²/g, and a total pore volume (V_total_) of 0.269 cm³/g, of which 0.010 cm³/g was attributable to micropores (V_micro_). The adsorption-based average pore diameter (APD) was 29.4 nm. Following dealkalization, S_BET_ increased slightly to 38.49 m²/g, V_total_ rose to 0.278 cm³/g, and the mesopore volume (V_meso_) increased from 0.153 to 0.208 cm³/g, while S_micro_ exhibited a marginal increase and the APD decreased slightly to 28.9 nm. Such modest enhancements in surface area and mesopore volume are consistent with previous findings for RM subjected to mild chemical or thermal modification [49,50,66]. Comparable broadening of the mesopore fraction has been reported for acidified and thermally treated RM, where partial restructuring of larger voids into mesopores was suggested [62,66]. Although the magnitude of improvement here is less pronounced than that achieved through more intensive treatments, such as high-temperature calcination or mechanical activation [67,68], the observed changes align with reports linking increased mesoporosity to improved adsorption and mass transport properties [64,69].

**Table 4:** Textural parameters of untreated and dealkalized RM from BET, t-plot, and BJH analyses.

Sample	S_BET_ (m^2^/g)	S_micro_ (m^2^/g)	V_total_ (cm^3^/g)	V_micro_ (cm^3^/g)	V_meso_ (cm^3^/g)	V_meso_/V_total_ (%)	APD (nm)
Untreated RM	36.58	20.50	0.269	0.010	0.259	96.3	29.41
Dealkalized RM	38.49	21.04	0.278	0.010	0.268	96.4	28.91

#### Phase transformation.

**Fig 7:**
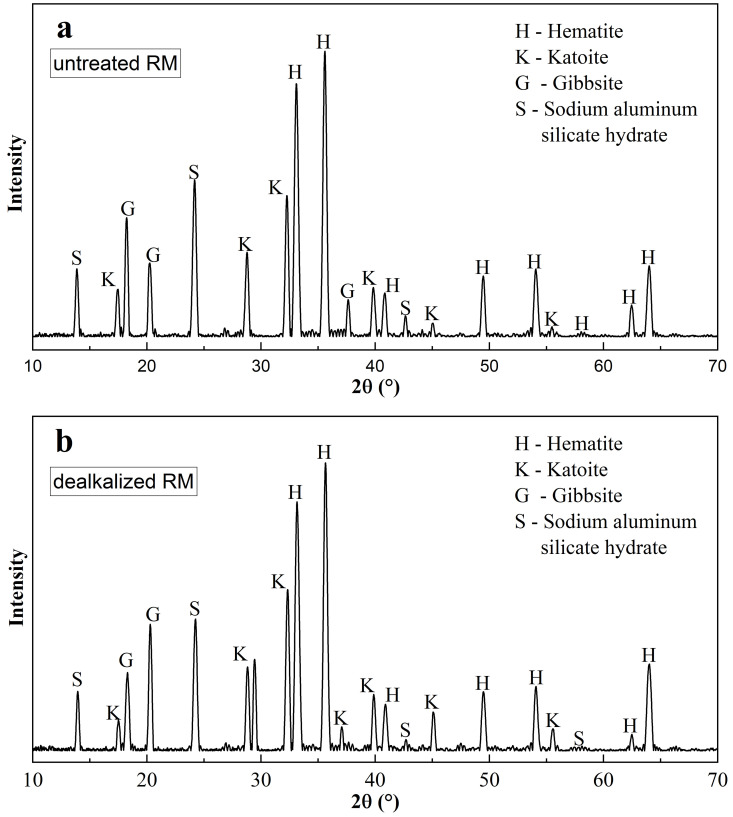
XRD patterns of RM: (a) untreated, (b) dealkalized RM.

The XRD patterns of untreated and dealkalized RM are depicted in Fig 7. The primary crystalline phases identified in both samples include hematite (Fe₂O₃), gibbsite (Al(OH)₃), katoite ((CaO)_3_(Al_2_O_3_)_1.75_(H_2_O)_3.75_), and sodium aluminum silicate hydrate ((Na_2_O)_1.08_·Al_2_O_3_·(SiO_2_)_1.68_·(H_2_O)_1.8_). Hematite, one of the dominant phases, showed no detectable alteration in its main reflections at 2θ = 33.15° and 35.61°, indicating preservation of crystalline integrity after dealkalization, in agreement with previous studies reporting its stability under mild washing conditions [5,27,40]. In contrast, the gibbsite peak near 18.26° exhibited a slight increase in intensity after dealkalization (Fig 7b), which may suggest enhanced crystal ordering or surface structural adjustment due to reduced Na⁺ interference in the lattice. Minor phases such as katoite and sodium aluminum silicate hydrate showed negligible variation, with their key peaks (e.g., 28.83°, 24.23°) remaining essentially unchanged (Fig 7b). Overall, the results suggest that dealkalization mainly affects surface-related structures and hydration states rather than causing substantial phase transformations.

### Effect of dealkalization on geotechnical properties

#### Specific gravity (G_S_) analysis.

The specific gravity (Gs) of the untreated RM was determined to be 2.87, which falls within the range typically reported for bauxite residues [70]. This comparatively elevated Gs value, relative to that of natural soils (typically 2.65–2.80) [71], may be related to the substantial presence of heavy metal oxides such as hematite, alumina, and titania, as indicated by XRD (Fig 7) and corroborated by XRF results (Table 3) [6,58]. Following dealkalization, Gs decreased slightly to 2.82, suggesting only marginal changes in particle density. Although Na₂O content showed a reduction after washing, the overall chemical composition appeared largely unchanged (Table 3), consistent with the absence of notable phase changes in XRD patterns (Fig 7) and only minor increases in surface area and mesopore volume observed in BET results (Table 4). This consistency suggests that the washing process primarily removed soluble salts without causing significant alterations in the chemical or physical structure of RM. In contrast, more intensive treatments reported in the literature—such as the nutrient-assisted microbial process used by Panda et al. (2017) [37]—have shown more pronounced changes in specific gravity, which may reflect a greater extent of mineralogical and chemical modification induced by such methods.

#### Grain size analysis.

Particle size characteristics of RM are known to vary depending on site-specific operational parameters, including ore mineralogy, digestion conditions, and clarification processes. Differences in alumina extraction methods, such as the Bayer and Sintering processes, can also lead to distinct particle size distributions, with Bayer-derived RM generally exhibiting finer textures [72]. Such variability is further influenced by the mechanical and chemical processes during refining, which determine the proportion of clay-, silt-, and sand-sized fractions [73].

**Fig 8:**
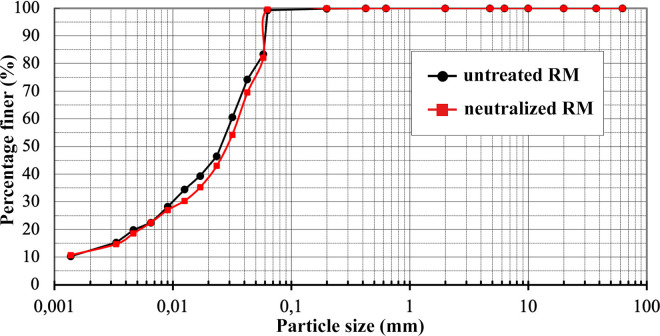
Grain size distribution curves of untreated and dealkalized RM determined via combined sieve and hydrometer analysis.

In this study, the grain size distribution of both untreated and dealkalized RM was determined using a combination of sieve and hydrometer analyses, as shown in Fig 8. The granulometry curve of the untreated RM indicates that 99.3% of the particles are finer than 63 µm, with approximately 12% classified as clay-sized and 87.3% as silt-sized fractions, while the sand fraction constitutes only 0.7%. The mean particle size (D₅₀) was approximately 25 µm, consistent with previously reported values for Bayer-derived RM, which is typically dominated by fine particles due to mechanical grinding and chemical separation during bauxite refining [71,73]. After dealkalization, a slight redistribution of particle sizes was observed, with the clay fraction decreasing to 11.9%, the silt fraction increasing to 87.7%, and the sand fraction declining to 0.4%, accompanied by a modest increase in D₅₀ to around 30 μm. This shift may indicate a limited degree of particle agglomeration, a tendency also reflected in SEM observations (Fig 5), which showed more cohesive and compact particle arrangements after treatment. BET analysis (Table 4) likewise revealed small increases in surface area and mesopore volume, which, while not conclusive, may be consistent with minor structural rearrangements that influence particle packing behavior. Similar behavior has been reported by Tsamo et al. [26], who observed increased particle size in RM after washing with distilled water, attributing this to interparticle interactions during the process. Lyu et al. [74] further demonstrated that neutralization treatments with seawater and gypsum promoted particle flocculation and aggregation through electrostatic bridging facilitated by multivalent cations such as Ca²⁺ and Mg² ⁺ . Likewise, Panda et al. [37] observed increased aggregation under bio-neutralization conditions, where microbial activity and associated biogeochemical reactions contributed to structural reorganization.

#### Atterberg’s limits.

The Atterberg limits of both untreated and dealkalized RM were determined to assess their plasticity characteristics, and the results are presented in Table 5. The liquid limit (LL) of the untreated sample was found to be 41%, the plastic limit (PL) 30%, and the plasticity index (PI) 11%, classifying the material as having low plasticity. These findings align with previous studies that report PI values typically ranging between 5 and 22 for RM, despite its lack of conventional clay minerals [75,76]. This atypical plastic behavior may be linked to the fine particle size and the presence of electrically active mineral phases identified in our XRD analysis (Fig 7)—notably hematite, gibbsite, katoite, and sodium aluminum silicate hydrate—which are considered influential in interparticle interactions and consistency behavior. Comparable mineralogical associations have likewise been documented in the literature for RM from different sources [77]. Following dealkalization, the LL increased to 46% and the PL to 34%, resulting in a modest rise in PI to 12%. Despite these changes, the plasticity classification of the RM remained unchanged, continuing to be categorized as a low-plasticity inorganic silt (ML) under the Unified Soil Classification System (USCS). Similar findings were reported by Panda et al. [37], who noted minor fluctuations in Atterberg limits following treatment, while the PI values remained within the low-plasticity range. Furthermore, source-dependent variability in RM’s plasticity behavior has been frequently documented and is largely linked to differences in mineralogical composition, industrial processing conditions, and the nature of residual ionic species retained within the particle matrix [70,78].

**Table 5:** Atterberg limits of untreated and dealkalized RM.

Sample	Liquid limit (%)	Plastic limit (%)	Plasticity index (%)
Untreated RM	41	30	11
Dealkalized RM	46	34	12

#### Compaction characteristics.

The permeability characteristics of both untreated and dealkalized RM were assessed using the falling head permeability test, with samples compacted at their respective MDD and OMC as determined from the standard Proctor test. The coefficient of permeability (k) for the untreated RM was measured at 7.24 × 10 ⁻ ⁶ cm/s, which is within the typical range reported for fine-grained soils such as silts and low-plasticity clays. This low permeability can be reasonably linked to the predominance of ultrafine particles and the relatively high specific surface area, which together promote a dense packing arrangement and limit fluid transmission. Similar values have been documented in previous studies on Bayer-derived RM, where hydraulic conductivity typically falls between 10 ⁻ ⁶ and 10 ⁻ ⁷ cm/s, largely independent of geographical origin or processing route [37,71,72]. The limited presence of coarse-grained constituents and the platy morphology of certain mineral phases, notably gibbsite and hematite as identified in our XRD analysis (Fig 7), may also contribute to this behavior.

**Fig 9:**
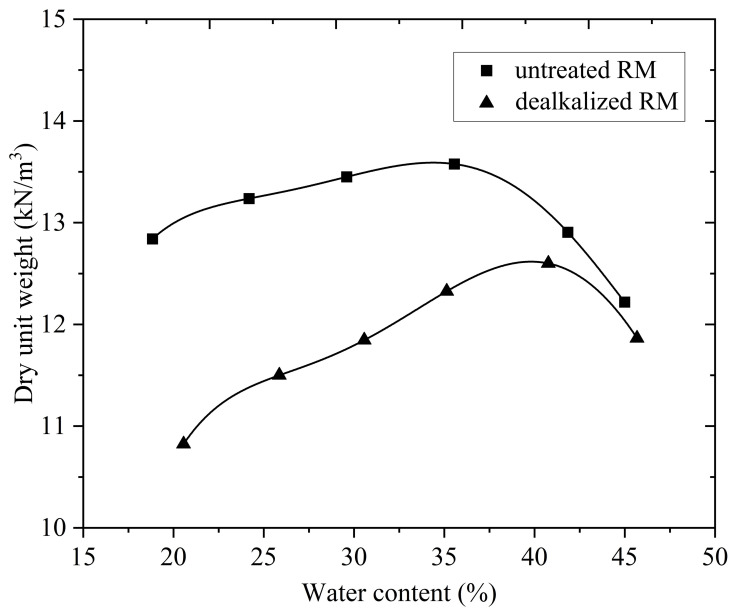
Standard Proctor compaction curves for untreated and dealkalized RM.

The compaction characteristics of both untreated and dealkalized RM were assessed using the standard Proctor test, with the resulting compaction curves shown in Fig 9. The untreated RM exhibited a maximum dry density (MDD) of 13.6 kN/m³ and an optimum moisture content (OMC) of 33.6%, values typical of fine-grained, low-plasticity soils that generally display lower dry unit weights and higher moisture demand compared to natural fine soils. Following dealkalization, the MDD decreased to 12.6 kN/m³, while the OMC increased to 39.2%, indicating a shift in compaction behavior. This alteration may be linked to increased water-holding capacity and the formation of larger particle aggregates, both of which reduce packing efficiency under compactive effort. Such tendencies are consistent with SEM observations (Fig 4) showing more cohesive particle arrangements and with BET results (Table 4) revealing small increases in surface area and mesopore volume, which—while modest—may facilitate greater moisture adsorption during compaction. Similar effects have been reported by Panda et al. [37] and Reddy and Rao [75], where treatments modifying RM surface chemistry or particle structure, such as bio-neutralization or chemical conditioning, led to reduced MDD and elevated OMC. Furthermore, as highlighted by Yashmin and Sinha [70], compaction characteristics of RM remain highly variable across sources, with reported MDD values ranging from 15.5 to 18.5 kN/m³ depending on particle size distribution, mineralogy, and inherent moisture content.

#### Permeability characteristics.

The permeability characteristics of both untreated and dealkalized RM were evaluated using the falling head permeability test, with specimens compacted at their respective MDD and OMC as determined from the standard Proctor test (Fig 9). For the untreated RM, the coefficient of permeability (k) was 7.24 × 10 ⁻ ⁶ cm/s, which is within the range typically reported for fine-grained soils such as silts and low-plasticity clays [79–81]. This low permeability can be reasonably linked to the predominance of ultrafine particles (Fig 8) and the relatively high specific surface area (Table 4), which together promote a dense packing arrangement and limit fluid transmission [82]. The mineralogical composition, particularly the abundance of hematite and gibbsite observed in XRD (Fig 7), may further contribute to this behavior through their plate-like and angular morphologies, which favor close particle contact and reduced pore connectivity [71]. These results are consistent with previous studies, which have reported hydraulic conductivity values for untreated RM typically ranging between 10 ⁻ ⁶ and 10 ⁻ ⁷ cm/s, regardless of its geographic origin or processing method [37,70,71].

Following dealkalization, the hydraulic conductivity increased markedly from 7.24 × 10 ⁻ ⁶ to 5.99 × 10 ⁻ ⁴ cm/s, representing nearly two orders of magnitude higher permeability compared to the untreated RM. This substantial increase can be attributed to several interrelated microstructural changes induced by the washing process. Particle size distribution analysis (Fig 8) revealed a reduction in the clay-sized fraction accompanied by a relative increase in the silt-sized fraction, which likely resulted in a looser packing arrangement and improved pore connectivity. SEM images (Fig 4) further illustrated a tendency for particle aggregation to form larger clusters with more prominent interparticle voids, consistent with a less compacted microstructure. This interpretation is supported by the compaction test results (Fig 9), where dealkalized RM exhibited a lower MDD (12.6 kN/m³) and higher OMC (39.2%) compared to the untreated RM (13.6 kN/m³ and 33.6%, respectively). Such changes in compaction behavior are indicative of increased void ratios and altered particle–water interactions, both of which are likely to facilitate greater flow through the soil matrix. Similar behavior was also reported by Nie et al. [83], who observed that acid neutralization of RM led to enhanced particle aggregation and the formation of macroaggregates, thereby increasing water permeability.

#### Unconfined compressive strength.

**Fig 10:**
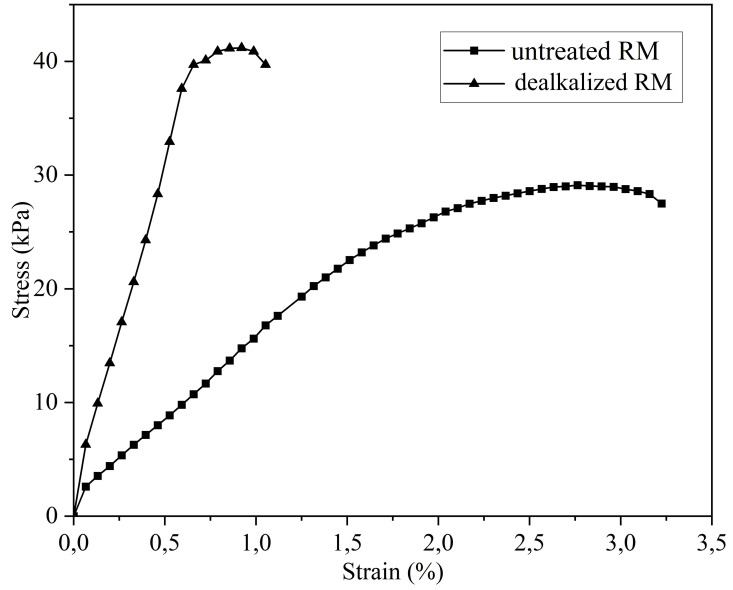
Stress-strain curve for untreated and neutralized RM from UCS tests.

The unconfined compressive strength (UCS) results for both untreated and dealkalized RM are presented in Fig 10. The untreated RM exhibited a UCS of 29 kPa, whereas the dealkalized RM reached 41 kPa, indicating a measurable improvement in strength following the washing process. This enhancement may be related to microstructural rearrangement induced by dealkalization. SEM observations (Fig 3) revealed that the dealkalized RM contained more densely clustered particle groups and macroaggregates compared to the untreated material. Such clustering can increase the number and area of interparticle contacts, potentially enhancing load transfer pathways. Results from the compaction tests (Fig 9) revealed a lower MDD and a higher OMC for the dealkalized RM compared to the untreated RM. Such changes would normally be associated with reduced strength in conventional fine-grained soils [84,85]. In the present case, however, these changes may indicate modifications in particle packing and pore structure. Along with possible changes in particle bonding, these factors could have helped offset the typical weakening effect of lower density, resulting in the observed strength gain. Previous studies have highlighted that RM often exhibits mechanical responses that deviate from those typically observed in natural fine-grained soils. For example, Newson et al. [77] reported that RM may show compressibility similar to clays while displaying frictional properties more akin to sandy soils, thereby highlighting the atypical combination of mechanical characteristics that distinguish this material. In this context, it should be emphasized that while the UCS values were clearly established, this explanation represents a plausible account based on the available evidence, and further targeted studies are required to substantiate the underlying mechanisms.

#### Free swelling behavior.

**Fig 11:**
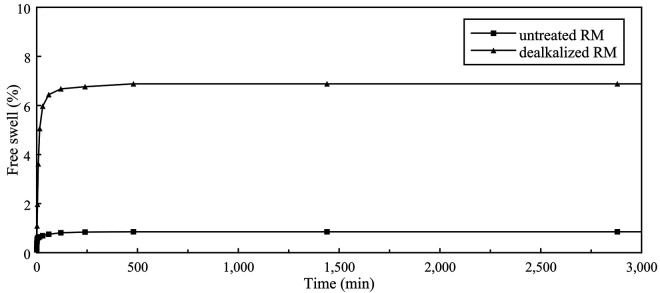
Free swell percentage of untreated and dealkalized RM.

One-dimensional free swell tests were performed on both untreated and dealkalized RM samples compacted at their respective MDD and OMC, with the free swell percentages presented in Fig 11 and the corresponding swell pressures summarized in Table 6. The untreated RM exhibited a very limited swelling response, with a free swell percentage of 0.85% and a swell pressure of 6.50 kPa, confirming its classification as a non-expansive material under saturated conditions. This limited swelling behavior is consistent with the measured Atterberg limits (Table 5) and the mineralogical composition revealed by XRD (Fig 7), which shows an absence of expansive clay minerals such as montmorillonite, thereby indicating a low capacity for interlayer water uptake and supporting the negligible swelling potential of RM. These results are in line with previous studies, which also reported that RM exhibits negligible swelling potential compared to conventional expansive soils [71,78,86,87].

**Table 6:** Swelling response of untreated and dealkalized RM.

Sample	Free swell (%)	Swell pressure (kPa)
Untreated RM	0.85	6.50
Dealkalized RM	6.90	25.60

In contrast, the dealkalized RM exhibited a pronounced increase in swelling, with a free swell percentage of 6.90% and a swell pressure of 25.60 kPa. This change is likely linked to the agglomeration of particles observed in SEM images (Fig 4b), which may have facilitated enhanced water ingress and retention. The increase is also consistent with the higher surface area and porosity measured by BET analyses (Table 4), both of which provide additional pathways for water uptake. In addition, the slight reduction in CaO content observed after dealkalization (Table 3) may have provided only limited support to the swelling increase, consistent with the suppressive role of CaO reported by Wang et al. [88].

## Conclusion

This study examined the effects of reagent-free dealkalization—performed through distilled-water washing—on red mud (RM) from the Seydişehir Eti Aluminum plant in Turkey, using an integrated chemical, mineralogical, morphological, and geotechnical assessment. The main outcomes derived from this study can be outlined as follows:

- Dealkalization lowered the pore-fluid pH from 10.65 to 9.35 and reduced soluble alkalis (Na₂O decreased from 6.00% to 4.36%, K₂O from 0.19% to 0.12%), while the major structural oxides remained essentially stable. These results indicate that the treatment selectively removed free alkalis without altering the overall chemical composition.
- XRD analysis revealed that the principal crystalline phases—hematite, gibbsite, katoite, and sodium aluminum silicate hydrate—remained essentially unchanged following dealkalization, with only a slight increase observed in the gibbsite peak intensity.
- SEM images indicated that dealkalization promoted denser particle agglomeration, reflecting a tendency toward microstructural rearrangement. EDS spectra supported these observations by confirming the persistence of major elements (Fe, Al, Si, Na, Ca, Ti) with no substantial compositional shifts after treatment.
- N₂ adsorption–desorption results indicated mesoporous characteristics in both samples, with the dealkalized RM showing a slight increase in surface area and mesopore volume.
- The specific gravity of untreated RM was 2.87, slightly decreasing to 2.82 after dealkalization, indicating only marginal changes in particle density.
- RM was predominantly composed of fine particles, with over 99% passing through the No. 200 sieve. Following dealkalization, the silt fraction increased slightly while the clay-sized fraction decreased. The specific gravity decreased marginally from 2.87 to 2.82.
- Both untreated and dealkalized RM were classified as ML (low-plasticity silt) according to the Unified Soil Classification System. Although liquid and plastic limits increased slightly after treatment, the plasticity index remained low, consistent with the behavior of RM as a non-expansive fine-grained material.
- Dealkalization reduced the maximum dry density from 13.6 to 12.6 kN/m³ and increased the optimum moisture content from 33.6% to 39.2%. Permeability rose markedly from 7.24 × 10 ⁻ ⁶ to 5.99 × 10 ⁻ ⁴ cm/s, likely reflecting the development of larger voids associated with particle clustering.
- The untreated RM exhibited a UCS of 29 kPa, which increased to 41 kPa after dealkalization. This strength gain may be related to particle clustering and enhanced interparticle contacts observed in SEM.
- The untreated RM showed negligible swelling (0.85%, swell pressure 6.50 kPa), consistent with its non-expansive character. After dealkalization, the free swell increased to 6.90% and swell pressure to 25.60 kPa, which may be associated with changes in porosity and particle aggregation that appear to facilitate greater water uptake.

## Supporting information

S1 Data Minimal dataset. (XLSX)
